# A conversation with Dr. Robert F. Kushner, MD, Professor of Medicine (Endocrinology) and Medical Education, Northwestern University Feinberg School of Medicine

**DOI:** 10.1017/cts.2022.518

**Published:** 2023-02-15

**Authors:** Kathy Siranosian

**Affiliations:** Clinical Research Forum, Washington, DC, USA

## Top 10 Clinical Research Achievement Awards Q & A

This article is part of a series of interviews with recipients of Clinical Research Forum’s Top 10 Clinical Research Achievement Awards. This article is with Dr. Robert F. Kushner, MD, Professor of Medicine (Endocrinology) and Medical Education, Northwestern University Feinberg School of Medicine. Dr. Kushner studies nutrition and weight management with a focus on medical care and preventative medicine for patients who are overweight or have obesity. Dr. Kushner received a 2022 Top 10 Clinical Research Achievement Award for *Once-Weekly Semaglutide in Adults with Overweight or Obesity* [1]. *The interview has been edited for length and clarity.*

## When Did You First Get Interested in Clinical Research?

After my residency, I did a 2-year combined fellowship and master’s degree in clinical nutrition, and part of that training program was to do clinical research. That was actually my first experience conducting my own research and I found it very exciting from start to finish – from planning out the project and developing hypotheses to conducting the study, analyzing the data, and going through the publication process. Clinical research is powerfully enriching and rewarding because it culminates in a product that not only you can see, but that others are also able to see and benefit from.

## How Has the Field of Clinical Nutrition Changed Since Then?

I started my practice roughly 40 years ago and since then, clinical nutrition has evolved tremendously. For example, over that time, obesity became the most prevalent chronic disease in our society, and I began focusing on it more and more. I helped found the American Board of Obesity Medicine, and now, there are multiple areas of research aimed at helping patients who are overweight reduce body weight and improve overall health. Our award-winning trial looked at one approach. We showed that among adults with overweight or obesity (without diabetes), once-weekly subcutaneous semaglutide plus lifestyle intervention was associated with substantial, sustained, clinically relevant mean weight loss.

## Is This Type of Adjunctive Pharmacotherapy the Direction Obesity Medicine Is Headed?

The medications that are on the horizon for reducing weight are incredibly exciting and they’re ushering in a paradigm shift in obesity care. It’s really been only in the past decade or two that we’ve begun to understand what’s going on with obesity from a biologic point of view, and that’s enabled pharmaceutical companies to begin to synthesize versions of the molecules that come from the gut and regulate appetite. Our paper was about just one of the many emerging compounds in this field, so stay tuned because there is going to be more. In the past, the FDA was hesitant about moving these drugs forward, but that’s changing, which is good news. Remember, 42% of the American population suffers from obesity.

## Diet and Exercise Have Long Been the Cornerstone of Weight Management. Could Medications Eventually Replace Lifestyle Interventions?

That’s a very interesting question. To answer it, let’s look at the changes we’ve seen with another health risk: smoking. We used to train medical students and residents for hours and hours on smoking cessation counseling. But today, because there are effective pharmacological treatments that can be offered to patients instead of, or in addition to, counseling, we do not have to spend as much time on smoking cessation counseling. Granted, obesity care is not quite as simple – it’s not just one behavior; it’s a disease with many determinants – but as we develop more effective medications, we are entering a new generation of patient care. I wouldn’t say medications will replace lifestyle counseling completely, but they can make lifestyle counseling easier. Plus, patients will be able to adhere more consistently and successfully because these biological treatments govern appetite and guide healthier eating.

## Are There Other Ways You Anticipate Patient Care Changing?

I’m also very involved in educational research, and I am working with colleagues on improving the curriculum for medical school residents in this area. Unfortunately, as it stands now, we are graduating medical students and residents who do not have sufficient competency in caring for patients struggling with their weight. Considering weight management is one of the most common problems patients have, we really have to do a better job at educating doctors on how to help patients. They need to learn how to use empathy and communication skills to build trust so that patients will open up about their concerns. As an example, in part of my education research, we developed a series of competencies for learners about obesity care, from epidemiology to science to practice. Our research involved the development of a simulated encounter with a standardized patient where medical residents take an obesity history and then undergo a validated assessment of their skills. These skills can be applied to patient care across other areas, as well.

## What Advice Do You Have for People Considering a Career in Clinical Research?

First, choose a career path that best suits your interest and passion. You do not want to get caught up in something because someone else wants you to do it, and that means you need to be clear about your path. Second, understand what you can realistically accomplish so that you do not overcommit. Third – and this trumps the others – identify a supportive and experienced mentor who will guide your career. This is critical because without a mentor, you’ll be running in circles. You need someone who you trust to look up to someone who has your back early in your career and can make sure that you are moving forward and being as productive as possible. I was fortunate that I had a mentor very early during my fellowship and master’s program. The field of nutrition is huge and my mentor was the one who helped me marry nutrition and obesity at the start of my career.

## How Do You Stay Motivated?

It’s extremely rewarding to work in this field and to be involved in what is next. I’m motivated because I see the dramatic impact weight loss can have on people’s lives. It’s a metamorphosis. It opens up things that they never thought they could do – being more active, traveling, hiking, or simply just crossing their legs. It’s amazing how it changes people, and it’s such a common problem.
